# Mechanisms underpinning use of new walking and cycling infrastructure in different contexts: mixed-method analysis

**DOI:** 10.1186/s12966-015-0185-5

**Published:** 2015-02-21

**Authors:** Shannon Sahlqvist, Anna Goodman, Tim Jones, Jane Powell, Yena Song, David Ogilvie, on behalf of the iConnect consortium

**Affiliations:** Centre for Physical Activity and Nutrition Research (C-PAN), School of Exercise and Nutrition Sciences, Deakin University, 75 Pigdons Rd, Geelong, Australia; Medical Research Council Epidemiology Unit and UKCRC Centre for Diet and Activity Research (CEDAR), University of Cambridge, Cambridge, UK; Faculty of Epidemiology and Population Health, London School of Hygiene and Tropical Medicine, London, UK; Department of Planning, Oxford Brookes University, Oxford, UK; Centre for Health and Clinical Research, University of the West of England, Bristol, UK; School of Civil Engineering and the Environment, University of Southampton, Southampton, UK

**Keywords:** Built environment, Context, Cycling, Evaluation, Infrastructure, Intervention, Mechanism, Mixed-method, Qualitative, Walking

## Abstract

**Background:**

Few studies have evaluated the effects of infrastructural improvements to promote walking and cycling. Even fewer have explored how the context and mechanisms of such interventions may interact to produce their outcomes.

**Methods:**

This mixed-method analysis forms part of the UK iConnect study, which aims to evaluate new walking and cycling routes at three sites — Cardiff, Kenilworth and Southampton. Applying a complementary follow-up approach, we first identified differences in awareness and patterns of use of the infrastructure in survey data from a cohort of adult residents at baseline in spring 2010 (n = 3516) and again one (n = 1849) and two (n = 1510) years later following completion of the infrastructural projects (Analysis 1). We subsequently analysed data from 17 semi-structured interviews with key informants to understand how the new schemes might influence walking and cycling (Analysis 2a). In parallel, we analysed cohort survey data on environmental perceptions (Analysis 2b). We integrated these two datasets to interpret differences across the sites consistent with a theoretical framework that hypothesised that the schemes would improve connectivity and the social environment.

**Results:**

After two years, 52% of Cardiff respondents reported using the infrastructure compared with 37% in Kenilworth and 22% in Southampton. Patterns of use did not vary substantially between sites. 17% reported using the new infrastructure for transport, compared with 39% for recreation. Environmental perceptions at baseline were generally unfavourable, with the greatest improvements in Cardiff. Qualitative data revealed that all schemes had a recreational focus to varying extents, that the visibility of schemes to local people might be an important mechanism driving use and that the scale and design of the schemes and the contrast they presented with existing infrastructure may have influenced their use.

**Conclusions:**

The dominance of recreational uses may have reflected the specific local goals of some of the projects and the discontinuity of the new infrastructure from a satisfactory network of feeder routes. Greater use in Cardiff may have been driven by the mechanisms of greater visibility and superior design features within the context of an existing environment that was conducive neither to walking or cycling nor to car travel.

**Electronic supplementary material:**

The online version of this article (doi:10.1186/s12966-015-0185-5) contains supplementary material, which is available to authorized users.

## Introduction

There is widespread acknowledgement that certain types of built environment design are more likely to support walking and cycling than others [1]. For example, surveys suggest that cyclists often prefer to cycle on segregated paths [2,3] and cross-sectional and ecological studies suggest that neighbourhoods more conducive to walking and cycling tend to have higher levels of these behaviours, although these associations may also reflect neighbourhood-selection bias or pre-existing demand for improvements in infrastructure [4-8]. The lack of more robust evidence for causal inference reflects the difficulty of manipulating environmental change for experimental purposes, such as in randomised controlled trials. Natural experimental studies that evaluate planned changes to the environment using quasi-experimental methods are therefore recognised as key to enhancing our understanding of the relationship between the built environment and walking and cycling [9,10].

A number of natural experimental studies have evaluated the effects of infrastructural improvements (such as the construction of new cycle routes) on walking and cycling [11-19]. Given the challenges inherent in conducting studies of this kind, it is not surprising that much of the existing research suffers from important methodological limitations such as a lack of comparison data [16,18] or a reliance on retrospective measures of physical activity [11] or simple counts of users [14,17]. Although previous studies have demonstrated that people used the infrastructure provided, few have examined broader population-level changes in walking and cycling relative to a comparison group. One study reported that among respondents aware of the infrastructure there was an increase in the proportion who had cycled at least once in the past year [20]. This and another study also reported increases in cycling among cyclists living near the infrastructure [12,20], whilst another study found that use of the infrastructure did not result in an increase in physical activity [15].

Even fewer studies have gone beyond evaluating whether an intervention was effective in increasing walking, cycling or overall physical activity (investigating *outcome*s) to explore in detail the *mechanisms* (putative causal pathways) underlying intervention effectiveness and the *context* (setting) in which an intervention was (or was not) effective [21]. For example, new infrastructure may be expected to increase walking and cycling (outcomes) by improving the safety and convenience of the route for a given journey (mechanism), but the effect may be conditional on the perceived convenience of car travel for that journey (context). Exploring the relationships between context, mechanisms and outcomes is central to the notion of *realistic evaluation* — an approach that appears particularly pertinent to the evaluation of infrastructural projects, which are typically highly specific to their context [22].

We therefore drew on aspects of the realist approach to evaluation in the design of the iConnect study. The study aimed to evaluate new, purpose-built infrastructure for walking and cycling constructed as part of Connect2, a programme of projects led by Sustrans (a sustainable transport charity) to build or improve walking or cycling routes in 84 communities around the UK [23]. Although each Connect2 project was unique, all of them had the common goal of ‘creating new routes for journeys we make every day’. It was hypothesised that the schemes would improve the accessibility of local destinations by improving the convenience, safety, pleasantness or other aspects of the routes to those destinations and that these changes would lead to increases in walking and cycling and wider changes in overall travel behaviour and physical activity [24].

Using aggregate survey data on awareness and use of the schemes as well as changes in walking, cycling and overall physical activity collected from cohorts of local residents at three Connect2 sites, we have previously shown that over one-third (38%) of respondents across all three sites reported using the new infrastructure at two-year follow-up. Respondents were also more likely to report using the infrastructure for walking than for cycling, and for recreation than for transport [25]. We have also shown that living closer to the infrastructure predicted increases in time spent walking, cycling and in overall physical activity at two-year follow-up [26]. In this paper we build on these findings; first by examining differences in awareness and patterns of use *between* sites, and second by seeking to explain these differences by integrating qualitative interview data from key informants with survey data on changes in perceptions of the environment among residents. This ecological mixed-method analysis of qualitative and quantitative data at the area (site) level aims to explore the mechanisms by which the schemes may have led to the promotion of walking and cycling and the settings or contexts that enabled (or prevented) these changes.

## Methods

### Study sites

The selected Connect2 project sites were Cardiff in south Wales, Kenilworth in the English Midlands and Southampton on the south coast of England. These three sites were selected for detailed investigation because they were accessible to researchers, were deemed likely to have a measurable population impact and provided some heterogeneity in content and context [23]. Further information on the three study sites is provided in Table 1, and maps detailing the core project and feeder routes at each site are provided in Additional file 1: Figures S1-S3).

**Table 1:** **Overview of the three case study sites**

Cardiff	The Cardiff project consists of five elements. The core infrastructural component is the Pont-y-Werin (People’s Bridge), a 140 m traffic-free pedestrian and cycle bridge. The bridge completes a 5 km circular link around Cardiff Bay, crossing the River Ely to connect Penarth and the Cogan Railway station to the city centre. It provides a route between Cardiff city centre and Cardiff Bay on one side and the suburbs of Penarth and Dinas Powys on the other side. The other four elements of the development were feeder routes to and from the bridge to facilitate access and use.
Kenilworth	There are two primary elements to the Kenilworth project including the upgrade and creation of approximately 10 km of dedicated walking and cycling paths and a new bridge crossing a busy dual carriageway (A429 Coventry Road). The first component of the route starts at Abbey Fields and follows a pathway behind a housing development before crossing minor roads and continuing through Kenilworth Common conservation area. The route then meets an existing greenway at the new pedestrian and cycle bridge spanning the A429 Coventry Road (second component). A third component, a separate path leaving the Greenway and crossing farmland northwards to the nearby university campus (known as the Green Corridor) was planned but not completed.
Southampton	The Southampton project, known as the River Itchen Boardwalk, comprises a raised walkway built on top of a wave wall. It provides a north–south connection through the city and is intended to connect local people to the river and sea. An informal footpath along the shore had been used by local residents to avoid long detours around a busy industrial estate, but the footpath was impassable at high tide and unsuitable for cyclists. The route is also linked with existing National Cycle Network routes.

The Connect2 projects had not been completed at the time of the baseline survey in April 2010. The core projects in Southampton and Cardiff opened in July 2010 (shortly after the baseline survey) and most feeder routes had been upgraded at all sites by the time of one-year follow-up. The completion of the core project at Kenilworth was delayed until September 2011.

### Interviews with key informants

In 2009, prior to the completion of the Connect2 schemes, semi-structured interviews were conducted with 17 key informants from relevant local and national stakeholder organisations including representatives of Connect2 local steering groups, local authorities, cycling groups, building contractors and Sustrans. Interviews were conducted prior to completion of the schemes, because their aim was to understand the context within which each scheme was implemented while avoiding the risk of bias or post-hoc rationalisation due to knowing what had actually occurred. Most interviews were conducted face-to-face although a few were conducted over the telephone. The topic guide included a series of open-ended questions to elucidate the background to the local Connect2 project or the overall Connect2 programme; which groups within the local population were thought likely to use the infrastructure, for what types of journeys, and to and from which areas and destinations; and whether the success of the project was dependent on other local factors such as feeder routes. Interviews were conducted by one lead researcher for each of the three case study sites and another for the national representatives. Audio recordings were made of the interviews and their contents were transcribed verbatim with the written informed consent of the participants.

### Core survey of residents

#### Sample

The core survey methods and questionnaire have been reported in detail elsewhere [23,24]. In April 2010, 22 500 adults living within 5 km by road of the planned Connect2 infrastructure were identified at random from the edited electoral register and sent a survey pack to complete. 3516 (15.6%) respondents returned a completed baseline survey and were re-surveyed in April 2011 and April 2012. In 2011, 1885 respondents returned a follow-up survey, of whom 36 had moved home and were excluded leaving 1849 for inclusion in analysis (53% retention). In 2012, 1548 of the original 3516 respondents returned a follow-up survey, of whom 38 had moved home, leaving 1510 for inclusion in analysis (48% retention). Participant characteristics are summarised in Additional file 1: Table S1 and have been described in more detail elsewhere [25]. Briefly, 54% of respondents were women, 13% were aged 18–34 years, 21% were aged 35–49 years, 33% were aged 50–64 years and 33% were aged 65 years or over. Participants were older on average than the general population of their local area, but the sample was otherwise broadly representative in terms of demographic, socioeconomic and travel-related characteristics [25].

### Measures

#### Awareness and use of Connect2

The follow-up questionnaires included items to ascertain awareness and use of the schemes. These were adapted for each study site and prefaced with a description of the relevant scheme. For example, in Southampton the question began *“You may be aware that in the past year a new pedestrian and cycle route has been opened between St Denys and Bevois Valley/Northam. This is known locally as the ‘Itchen Riverside Boardwalk’.”* Participants were asked to report their awareness (yes/no) and use (yes/no) of the scheme in general, followed by whether they used the infrastructure for walking or cycling for each of six purposes (yes/no).

#### Perceptions of the neighbourhood and route environments

At each survey wave, perceptions of the local neighbourhood (defined in the questionnaire as within a 10–15 minute walk from home) were assessed using a set of 13 items adapted from the ALPHA European Environmental Questionnaire [27]. All items were rated on a five-point Likert scale from ‘strongly disagree’ (coded as −2) to ‘strongly agree’ (coded as 2). These items were found to have acceptable test-retest reliability comparable with that of the items in the ALPHA questionnaire [28]. Seven of these items were repeated with a site-specific question stem that referred to the specific route or area served by the new Connect2 infrastructure.

### Analytical approach

#### Theoretical framework

Our analytical approach was guided by the theoretical framework outlined by Ogilvie and colleagues [24] which postulated that the schemes would influence walking and cycling by one or a combination of (a) improving connectivity, which in turn could be associated with improvements in the availability and accessibility of destinations as well as the convenience, aesthetics and safety of the routes to these destinations; and (b) improving the social environment through changes in the prevalence of walking and cycling and social norms and social support for those behaviours.

**Figure 1:**
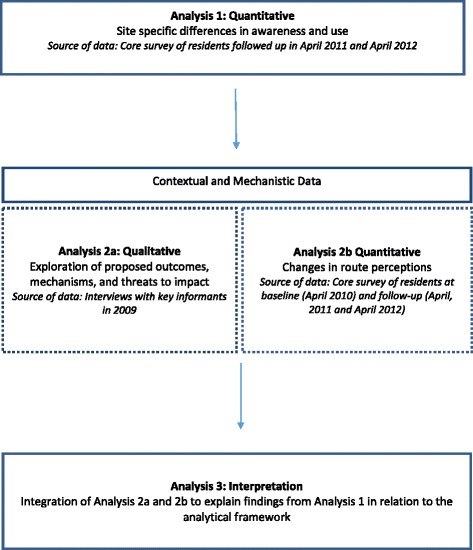
**Approach to data analysis.**

We took both a sequential and a parallel approach to analysis, as summarised in Figure 1 [29]. We first analysed the quantitative awareness and use data to determine any site-specific differences in use of the Connect2 schemes (Analysis 1). We then analysed, in parallel, the qualitative interview data on the context and hypothesised mechanisms of each Connect2 project (Analysis 2a) and the quantitative data on changes in residents’ perceptions of the environment at each site (Analysis 2b) to interpret the findings from Analysis 1 and explain any unexpected outcomes. We were guided by the priority sequence framework outlined by Morgan [30], applying the ‘complementary follow-up’ approach. As Greene and colleagues describe, complementarity seeks elaboration, enhancement, illustration and clarification of the results from one method with the results of another method [31]. Consistent with a follow-up approach, the quantitative data on awareness and use were viewed as the primary data source and the qualitative interview data and quantitative data on environmental perceptions as secondary, yet equal, data sources. This approach is particularly useful when smaller qualitative studies are used to help evaluate and interpret the results from a principally quantitative study [30]. Following Analysis 2, the two datasets were integrated at the interpretation phase to explain the findings from Analysis 1 as they related to the theoretical framework (Analysis 3, presented in the Discussion section) [30].

### Qualitative analysis

Using techniques described by Green and colleagues [32], qualitative analysis was led by SS with peer-checking by the relevant interviewers and lead investigators (TJ, YS, JP, DO). The purpose of the first phase of analysis was to identify the proposed outcomes, potential influences on these outcomes and the postulated mechanisms leading to these outcomes so these three broad concepts guided the analysis. After immersion in the transcripts, data were examined and organised (coded) and relevant categories created from these codes. As coding continued, each category was challenged by searching for any contradictory patterns. Later analysis (Analysis 3) focused on integrating the qualitative and quantitative route perceptions data to explain and interpret key findings in relation to awareness and use of the schemes. The entire process was validated by iterating the initial interpretations of the qualitative data with the other interviewers through discussion and revision of serial drafts.

### Quantitative analysis

Data on awareness and use of Connect2 by site were summarised using simple descriptive statistics. Data on route perceptions were also summarised by site and mean changes between 2010 and 2011 and between 2010 and 2012 were calculated for each item. To examine whether any changes in route perceptions reflected more general changes in residents’ perceptions of their neighbourhood, we also calculated site-specific mean changes for the six comparable neighbourhood perception items in residents living within 2 km of the infrastructure.

The University of Southampton Research Ethics Committee granted ethical approval for the interviews with key informants and the core survey (CEE200809-15).

## Results

### Survey data on awareness and use of the schemes (Analysis 1)

Although awareness of Connect2 was relatively high overall — 64% and 73% of participants reported having heard of it in 2011 and 2012 respectively — this varied substantially between sites. In Cardiff awareness was 86% in 2011 and increased slightly to 91% in 2012. In Kenilworth awareness was 57% in 2011 and increased more substantially to 71% in 2012, while in Southampton awareness was lower in both 2011 (47%) and 2012 (55%). Similarly, use of Connect2 varied substantially between sites; 49% of participants in Cardiff reported using the infrastructure in 2011, increasing slightly to 52% in 2012. By comparison, only 28% of participants in Kenilworth and 19% of participants in Southampton reported using the infrastructure in 2011; these proportions increased in 2012, more so in Kenilworth (to 37%) than in Southampton (to 22%).

Table 2 provides information on intervention awareness and use by site in 2012. Patterns of use were broadly similar in 2011 and are therefore provided in Additional file 1: Table S3. Across all sites, many more residents reported using the schemes for recreational purposes than for utility purposes. The distribution of modes and purposes of use was similar across the sites, except that Kenilworth showed a greater dominance of recreational over utility use and there was some evidence (although not statistically significant) that the Cardiff scheme was used more for commuting purposes.

**Table 2:** **Awareness and use of Connect2 infrastructure in 2012, by site, mode and purpose**

	**Use in 2012**			
	**% full sample (N = 1490)**	**%Southampton (N = 419)**	**% Cardiff (N = 482)**	**% Kenilworth (N = 589)**
Awareness or use of Connect2	73%	55%	91%	71%
Use of Connect2 (any)	38%	22%	52%	37%
Walking (any)	35%	19%	49%	34%
Transport (any)	12%	9%	21%	7%
Social/leisure	8%	6%	14%	5%
Shopping/personal business	6%	3%	11%	3%
For work	1 (2%^†^)	1 (1%^†^)	2 (5%^†^)	1 (1%^†^)
In the course of work	1 (1%^†^)	1 (1%^†^)	2 (3%^†^)	<1 (<1%^†^)
For education	1%	1%	1%	<1%
Recreation	32%	15%	46%	33%
Cycling (any)	16%	11%	22%	15%
Transport (any)	7%	6%	12%	4%
Social/leisure	5%	4%	8%	3%
Shopping/personal business	2%	3%	4%	1%
For work	2 (4%^†^)	1 (2%^†^)	4 (7%^†^)	1 (2%^†^)
In the course of work	1 (2%^†^)	1 (2%^†^)	1 (3%^†^)	<1 (1%^†^)
For education	<1%	<1%	1%	<1%
Recreation	15%	8%	21%	15%
Any walking or cycling	37%	22%	52%	37%
Transport (any)	14%	11%	24%	8%
Social/leisure	10%	8%	16%	6%
Shopping/personal business	7%	4%	13%	3%
For work	3% (5%^†^)	1% (3%^†^)	5% (10%^†^)	2% (2%^†^)
In the course of work	1% (2%^†^)	1% (2%^†^)	2% (4%^†^)	1% (1%^†^)
For education	1%	1%	1%	<1%
Recreation	34%	17%	49%	35%

Analyses exclude 1.3% adults with missing ‘use’ data in 2012.

^†^Percentage of those who reported being in paid employment.

### Interviews with key informants (Analysis 2a)

The main categories emerging from the interview data concerned the expected use and impact of the schemes, the perceived need for them, their visibility, the scale of environmental change they entailed and their design features. Example quotes are provided in the text and in Table 3, with additional quotes provided in Additional file 1: Table S2. To avoid inadvertently disclosing the identities of the participants, exact details of their organisations and job titles are not reported, but each participant is identified by a code indicating their membership of one of the four groups of informants (C1-4: Cardiff, K1-6: Kenilworth, N1-3: national and S1-4: Southampton).

**Table 3:** **Informants’ quotes illustrating categories**

**Cardiff**	**Kenilworth**	**Southampton**
**Expected use and impact**		
**Utility perspective**	**Utility perspective**	**Utility perspective**
The bridge will enable commuters to travel from Penarth into Cardiff and vice-versa, trips for leisure to the sports facilities, ice rinks, shops and other Bay facilities… (C3)	The main commute is going to be the lecturers and other staff at the university who live in Kenilworth. (K1)	…and as I said before, business, people going to work, to college, to the university…(S2)
**Recreational perspective**	**Recreational perspective**	**Recreational perspective**
The bridge will enable……. people to go on leisure rides. (C3)	The main use will be recreational. But there is a link that we’re always pushing between recreational sites and then cycling as the transport choice, it’s helpful if folks grow up cycle-friendly and know how to make the wheels go around and that’s the first step for any of us, getting on a bike. And also somewhere for people who used to cycle that are trepidatious about the roads and understandably, so re-finding their cycling legs. (K1)	I think people will use it because it’s along the river and the river is a big attraction for people to just, you know, use it for recreational purposes. (S3)
**Perceived need for the schemes – utility**		
**Challenges faced by pedestrians and cyclists**	**Challenges faced by pedestrians and cyclists**	**Challenges faced by pedestrians and cyclists**
Currently there are three routes to get into Cardiff from Penarth. None of these routes are user friendly for pedestrians and cyclists. (C1)	It will give mainly a traffic-free and it will give a continuous option whereas at the moment what exists is broken at Crackley [area] and it’s then severed at Gibbet Hill and you can’t actually access traffic free; this road [existing segregated route alongside the A429] is quite undulating, it’s got a couple of hills, they’re not steep but they might put some people off. This [the Connect2 infrastructure] does have a rise in it, but it’s gentler than that. (K5)	At the moment it, it feels very surrounded by busy road that area of St Denys, so having that nice link out is going to, I think it’s going to make people feel “oh yeah, you know, I could go on a cycle ride”. (S4)
**Existing options for walking and cycling**	**Existing options for walking and cycling**	**Existing options for walking and cycling**
Currently cyclists and pedestrians can use the Barrage, but this route doesn’t go into the Bay, it goes into east Cardiff. (C4)	I think [there will be a migration of existing users from the A429 route to the Connect2 route] certainly for getting to the main campus yes, for getting to Gibbet Hill campus, no. I think the most direct route will be up to the lights and down the Kenilworth Road. (K3)	Not identified
**Challenges faced by other road users**	**Challenges faced by other road users**	**Challenges faced by other road users**
The Cogan Spur, Penarth to Cardiff Bay, is always busy and has queues of traffic at all times of the day. (C2)	…and as I say, ties in with other initiatives you know [e.g. removing free parking], in terms of increasing or decreasing the incentive to drive on to the campus. (K3)	Not identified
**Perceived need for the schemes – recreation**		
Lots of people currently complain that they can’t get around the bay, so the bridge will be that missing link for circular walks and cycle rides around the bay. (C4)	…it’s a fantastic place for people with their young kids to go out, let them learn to wobble and fall off in a safe environment, which actually in a town like Kenilworth … it’s very difficult to find those places actually. (K5)	….there is a green area on the river that will now have a bench so it will be beneficial to people to sit and view the river; there are the views, visiting it to access the waterfront. (S1)
**Scale of environmental change**		
The main problem is that those who live near Penarth Town Centre, they don’t have a good route to the bridge. The Windsor Road from Penarth Town Centre is very narrow and extremely busy. It’s not a nice road to cycle along and doesn’t feel safe. (C2)	At the moment there is still a problem when you get to the Gibett Hill Traffic lights and turn left on their [leaving the A429 segregated shared walking & cycling path]. (K3)	I think there will be an improvement but it is not going to be in measurable terms. I don’t think it’s going to be huge, I think it’s going to be fairly marginal… (S4)
**Design features of the schemes**		
The bridge will have integral lighting which will make it safe for vulnerable groups at night. (C3)	…because what you’d got before was a dust stone surface, and we’ve widened it and sealed it so that it’s clean. So you think there are small tangible benefits that a mum will push her pushchair down there, she won’t get the wheels covered in muck… (K6)	The current development has a promenade around the perimeter, on the waterside, and there’s antisocial behaviour and damage that occurs there, and they were concerned that the boardwalk would increase the antisocial behaviour. (S1)

#### Expected use and impact of the schemes

In Cardiff, informants agreed that the new infrastructure would mostly be used for ‘*people to commute into the Bay and Cardiff [City Centre]*’ (C1). It was also regarded as important for people wanting to travel to and from the Bay’s sports and shopping facilities. Given that the bridge connected with existing infrastructure, stakeholders also believed that it would be used for recreational journeys.

Although the initial motivation for the Kenilworth scheme was to provide a walking and cycling route from Kenilworth to the nearby University of Warwick, informants’ expectation was that it would provide residents with a high-quality recreational resource. One explained that ‘*it goes across some very beautiful countryside and it just allows people to explore a wider area and pull together some interesting sort of leisure walks or bicycle rides’* (K6). In this regard, the scheme was recognised as ideal for introducing or reintroducing people, particularly children, to cycling. This view was consistent with expectations for the broader Connect2 programme, with informants expressing the view that an introduction to cycling was important to foster the growth of utility cycling in the long term. It was also suggested that people might use the scheme for general utility journeys such as shopping trips, albeit to a lesser extent. From a utility perspective, ‘*[The green corridor in the north of Kenilworth] will enable shorter journeys, more local traffic, you’ll get school runs on that, you’ll get people going to the shops, nipping to the [Kenilworth] town centre’* (K5).

The Southampton scheme was seen as benefitting both recreational and transport users, mainly because it formed an important link with existing infrastructure that was used for both utility and recreational purposes. One informant explained that ‘*as it’s part of the national cycle route, which a lot of cyclists and walkers like to do, it will fit in with that*’ (S1). The riverside location was seen as part of the scheme’s recreational focus, but on the other hand informants acknowledged that the scheme would service commuters and those wanting to travel to and from the airport and the university.

While informants acknowledged the importance of all three schemes for encouraging walking and cycling, emphasis was placed on an anticipated impact on cycling. There was a sense that cyclists, in particular, needed safer routes on which to travel and that suitable routes were currently lacking at each site. Moreover, the provision of high quality facilities was seen as important for encouraging those who had never cycled to take up cycling, and those who had given up to start cycling again.

#### Perceived need for the schemes

Informants agreed that the Cardiff bridge was built in response to the high volume of traffic common to the areas of Penarth and Cardiff Bay and was regarded as providing a viable alternative for non-motorised modes of transport. ‘*Traffic from Penarth to the Bay is dreadful*’ (C2), one informant explained, while others described parking difficulties and inefficiencies in the public transport system for those wanting to travel between the city centre and the west. Existing routes for those wanting to cycle into the Bay and city centre were described as hostile and unsafe, and walking was prohibited: *‘None of these routes are user friendly roads for pedestrians and cyclists’* (C1). The bridge was therefore thought likely to increase the ‘*number of people walking or cycling who previously used other means of transport*’ (C3). While an alternative longer route for cyclists and pedestrians did exist — the Cardiff Bay Barrage, opened in 2001 — it was argued that the Connect2 scheme would provide a more direct link to the city centre: ‘*It will also be a shorter route than the Barrage for accessing Cardiff Bay’* (C2).

Informants said that Kenilworth lacked a high-quality recreational resource, particularly for cycling, and that the scheme to turn an informal walking track into a dedicated walking and cycling path would make it accessible to a variety of user groups including young children, mothers and older adults. As one stakeholder explained *‘… as a weekend leisure route and as an introduction to cycling it is going to be very, very important*’ ([K5). For access to the university, the scheme was regarded as providing a more direct, traffic-free route for pedestrians and cyclists that avoided many of the hills on the cycle track alongside the existing busy main road. Although the existing route was described as unpleasant and hilly (Table 3), it was nevertheless regarded as of a relatively high standard and as providing a relatively direct continuous route. Concurrent changes to the university’s travel plan, including a reduction in car parking space and the removal of free parking, were expected to discourage motor vehicle use and increase demand for walking and cycling infrastructure.

In Southampton, the boardwalk was recognised as completing an important missing link. One informant explained that it ‘*will connect to the university via Portswood and it will be signposted up… it’s only 400 m long but it goes to many places*’ (S2). There were two existing routes — one through an industrial estate, which was described as unsafe due to heavy traffic, and one along an informal path, which was described as secluded and therefore giving rise to concerns about antisocial behaviour.

#### Visibility of the schemes

While the primary aim of Connect2 was to increase route connectivity and accessibility of local destinations, informants acknowledged that it also aimed to improve ‘journey literacy’ — in other words, to improve people’s knowledge of their locality to facilitate local journeys that were already possible. In this regard, the location of a scheme was seen as important. As one informant explained, ‘*there are some schemes that will be so visible that people will very quickly get it in to their mental map and that’s a phrase that’s bandied around here*’ (N1).

It was argued that for a scheme to be effective residents had to ‘*see, know, understand, get used to it’* and that this awareness *‘in some cases will happen instantaneously and in others it will be more gradual’* (N1). Informants believed that one way to improve journey literacy was by addressing the physical severance caused by linear geographical features such as rivers: bridging such obstacles was seen as creating ‘*living landmarks*’ (N1) capable of capturing the public imagination.

#### Scale of environmental change

Informants regarded the ‘coherence of routes’ as critical. It was thought that direct routes following desire lines and providing a continuous network were crucial for bringing about behaviour change. Nevertheless, informants raised concerns about the quality of feeder routes, particularly in Cardiff this was expected to have a substantial impact on the success of the project. Informants agreed that access would be hindered by aspects of road design (e.g. width and gradient) and concerns about personal safety (e.g. from heavy traffic). In Kenilworth the route lacked continuity for those wishing to travel to areas in the south of the town including the High Street, with users currently being required to continue on the main road using a route described as ‘*not for the faint hearted*’ (K3). Moreover, while it was always intended that the scheme would eventually link to the university, the completion of this section was delayed, forcing pedestrians and cyclists to use the separated cycle track along the main road but then to join a busy access road shared with motor traffic to enter the university campus. In Southampton, although specific concerns were not raised about the quality of the feeder routes, at only 400 m long the scheme was expressly viewed by some informants as being of an insufficient scale to bring about behaviour change.

#### Design features of the schemes

At each of the three sites informants commented on positive and negative design features of the schemes. In Cardiff, the width of the bridge and the fact that it was lit were recognised as crucial elements of design that ensured the scheme was safe and accessible to all. One informant commented that ‘*the bridge will have integral lighting which will make it safe for vulnerable groups at night’* (C3). Conversely, in Kenilworth, the scheme was unlit, passing through agricultural land. Moreover, in Kenilworth, one informant acknowledged that while the primary purpose of the Connect2 project was to encourage shorter journeys of less than two miles to be made by foot or on bike, the journey from Kenilworth to the university was substantially longer than this (5.5 km), which might limit its use in practice.

The Southampton scheme was built, in part, in response to concerns about crime and antisocial behaviour that was common along the route. Supporters of the scheme argued that it would encourage more people to use the route, which in turn would limit antisocial behaviour. On the other hand, one informant raised the possibility that the scheme might further encourage antisocial behaviour and that this would discourage its use.

### Survey data on perceptions of the environment (Analysis 2b)

#### Changes in route perceptions

**Figure 2:**
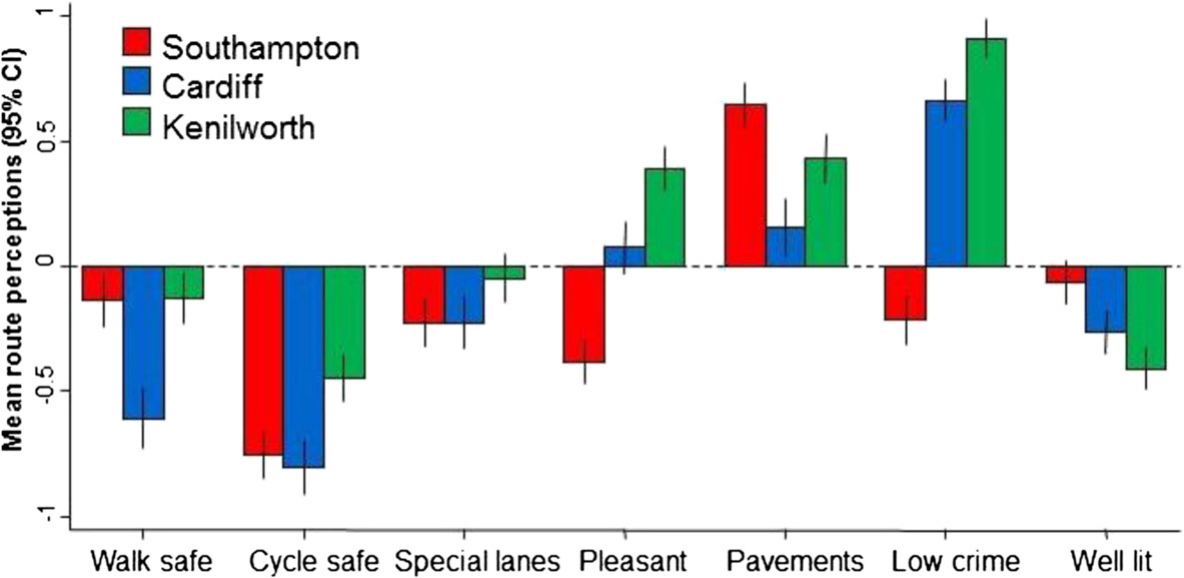
**Baseline perceptions of the routes altered by the Connect2 infrastructure.**

At baseline, perceptions of the routes to be served by the Connect2 schemes were generally unfavourable across all sites. This was particularly true for perceptions of cycling and walking safety and the presence of cycle lanes (Figure 2). Across all seven items, residents in Kenilworth appeared to have the most favourable baseline perceptions of the route, while those in Cardiff had the least favourable. The largest difference between the sites was for perceptions of safety from crime; mean perception scores for this item were positive in both Kenilworth and Cardiff, but negative in Southampton.

**Figure 3:**
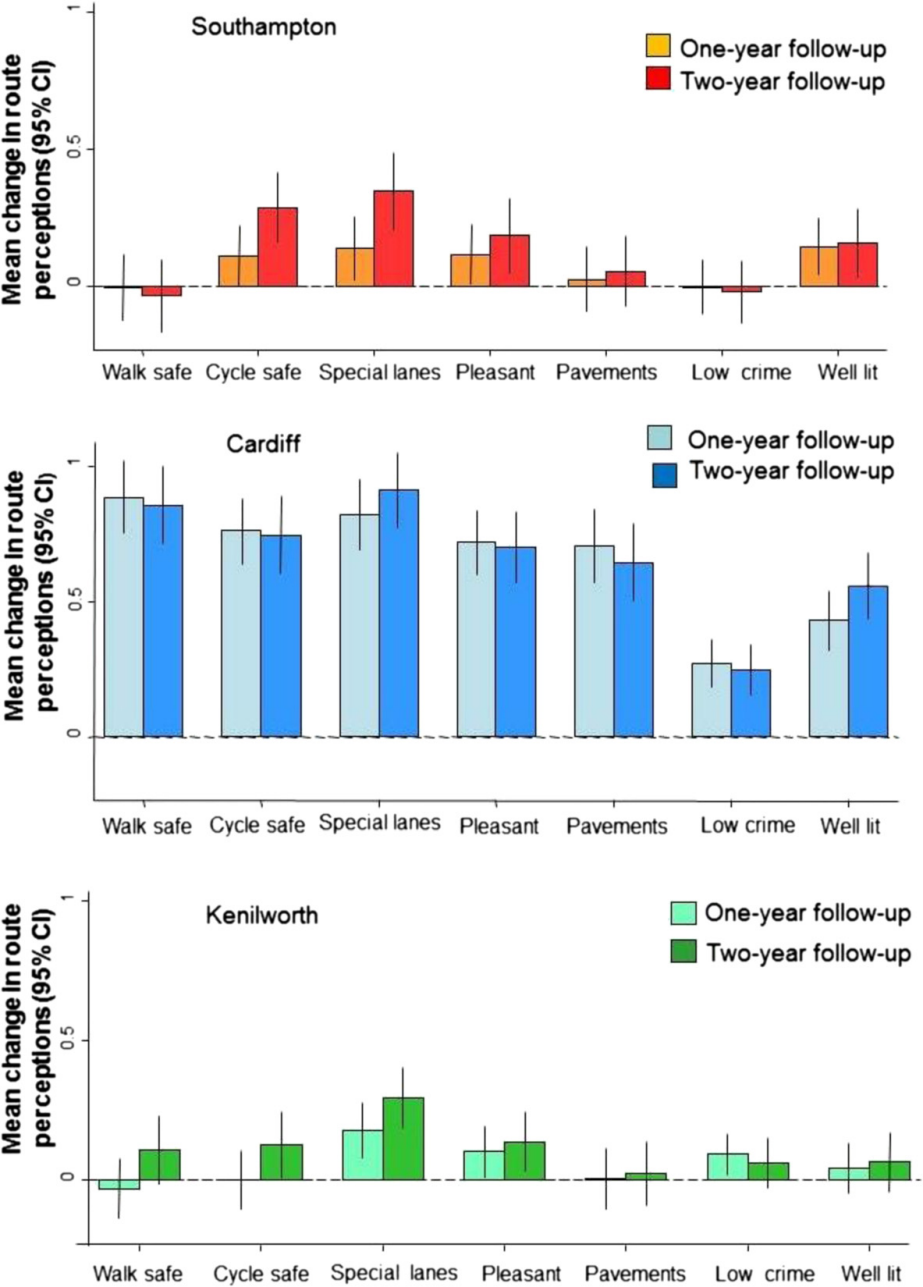
**Site specific changes in route perceptions between 2010 and 2011 and between 2010 and 2012.**

Figure 3 shows site-specific mean changes in perceptions of the routes. Among Cardiff respondents there were substantial improvements in all perception items between 2010 and 2011, and these improvements were maintained between 2010 and 2012. The increase was greatest for perceptions of walking safety and the presence of special lanes for cycling, and was smallest for perceptions of low crime, the latter plausibly reflecting the fact that perceptions of crime were already relatively favourable at baseline.

In Kenilworth and Southampton, improvements in route perceptions were less marked. In Southampton perceptions of the presence of cycle lanes and of cycling safety increased slightly between 2010 and 2011 and somewhat more between 2010 and 2012, as — to a lesser extent — did perceptions of the overall pleasantness and lighting of the route. In Kenilworth, the perception of the presence of special cycling lanes improved, as — to a lesser extent — did perceptions of the pleasantness of the route and the presence of pavements for walking. Marginal, non-significant changes were seen in the other items.

#### Changes in broader perceptions of the local neighbourhood

**Figure 4:**
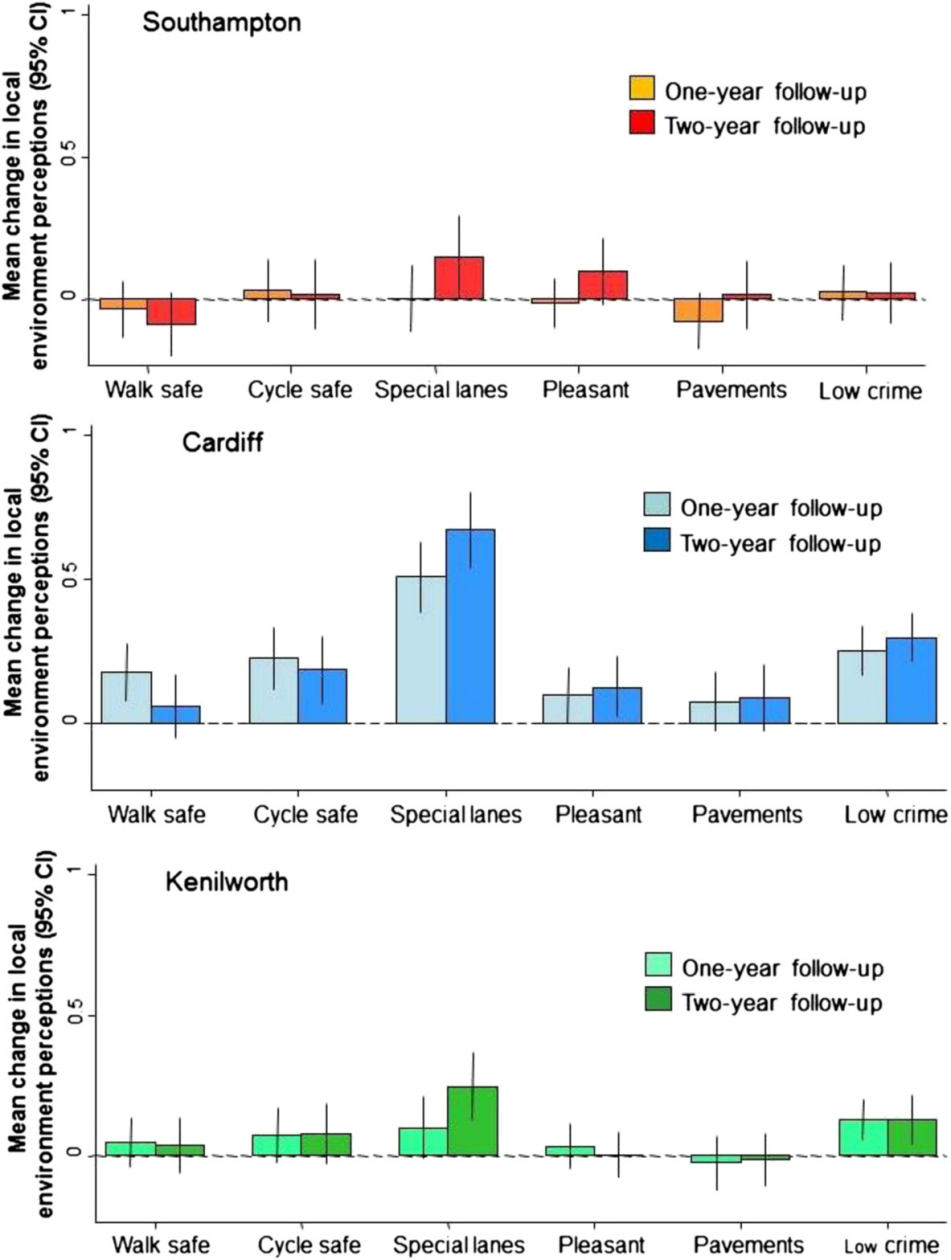
**Site specific changes in perceptions of the local environment between 2010 and 2011 and between 2010 and 2012 among individuals living less than 2 km from the core Connect2 project.**

The six comparable items applied to both the route and the local neighbourhood were then used to examine whether the changes in route perceptions were accompanied by changes in residents’ perceptions of their local neighbourhood in general (Figure 4). This appeared to be the case in Cardiff where increases in perceptions of the neighbourhood were seen for five of the six items, in particular for perceptions of the presence of special lanes for cycling. By contrast, in Southampton and Kenilworth there was little evidence that perceptions of the local neighbourhood improved, except for those of the presence of special lanes for cycling (at both sites) and of low crime (in Kenilworth).

## Discussion

Use of the infrastructure was substantially higher in Cardiff than in Kenilworth and Southampton, and residents across all three sites used the schemes primarily for walking and for recreational purposes. Synthesis of the qualitative and quantitative data provides insight into these patterns of use.

### Network-wide infrastructural improvements may be needed to promote utility walking and cycling

Despite the overarching goal of the Connect2 programme to ‘transform everyday local journeys,’ survey participants living near all three schemes were more likely to report using them for recreation than for transport. In Kenilworth in particular this appeared to reflect the fact that, despite initial aspirations to the contrary, the scheme was viewed as being mainly recreational in purpose. Across all three sites the dominance of recreational uses also appeared to reflect the need for network-wide improvements. Research suggests that continuous, dedicated walking or cycling routes appear to be more important for transport than for recreation [33], yet at all three sites informants acknowledged that users would still be required to navigate hostile walking and cycling environments on journeys involving the new routes. Alternatively, it may be that population density and land use mix are more important influences on active travel than the provision of specific infrastructure to increase connectivity [4].

It is also notable that survey respondents were less likely to report using the schemes for cycling than for walking. On the one hand this is perhaps not surprising and we have already argued that this is likely due to the higher prevalence of walking in general — almost five times more participants reported any walking in the past week compared with any cycling [25]. On the other hand, informants emphasised the importance of the schemes for promoting cycling — reflecting the view that the physical environment may be particularly important in determining the uptake of cycling [6,34,35]. It is possible that the poor quality of feeder routes had a greater impact on cycling than walking. Furthermore, findings from some stated preference studies suggest that the presence of safe routes may be important but not necessarily sufficient; individual fitness and the presence of end-of-trip facilities are among numerous other factors recognised to influence the uptake of cycling [3,36,37].

#### The visibility of infrastructural improvements may be important for generating awareness and use

The study has highlighted differences in awareness across the sites. The greater awareness in Cardiff may have been due to the scheme’s prominent positioning on a popular travel route, thereby ensuring that all residents — even those who never walked or cycled — were exposed to the infrastructure. By contrast, the Kenilworth and Southampton schemes were set back from main roads and less obvious to passers-by. Informants acknowledged the importance of visibility, arguing that schemes that were able to become part of residents’ mental maps would be effective in part by increasing awareness of other environmental supports. The survey data on route perceptions support this hypothesis somewhat: in Cardiff, not only did perceptions of the supportiveness of the route increase but so did those relating to the local neighbourhood in general, even among those who did not report using the Connect2 infrastructure (data not shown).

Creating visible schemes on desire lines may be important for generating awareness of new infrastructure. For less visible schemes, promotional strategies such as the use of media may be able to generate awareness. research suggests, however, the mere promotion of new infrastructure may be insufficient. For example, evaluation of the Cycling Connecting Communities intervention in Australia revealed that despite an extensive social marketing campaign, use of the cycling infrastructure increased by only 6% in the intervention community compared with 1% in the comparison community [20].

It may be that frequent visual exposure to a scheme is more important than awareness that it exists: this may provide not only knowledge of a safe more convenient route, but also reminders of the target behaviours and opportunities to make social comparisons with others using the scheme [38]. Indeed, social norms – the standards against which the appropriateness of a certain behaviour is assessed - are recognised as one of the most powerful forms of social control over human action [39]. Research to date suggests that descriptive norms (individuals’ perceptions of the prevalence of others’ behaviours) in relation to friends are an important influence on leisure-time physical activity [40,41] and that messages promoting descriptive norms may influence incidental physical activity [42]. Future research should explore the extent to which environmental interventions may bring about behaviour change by influencing psychosocial mechanisms of this kind.

#### The design of infrastructure may be important

The substantial differences in use between the sites may have also been driven by differences in important design features. The quantitative data on route perceptions revealed large positive changes in perceptions of the route in Cardiff compared with much smaller changes in Kenilworth and even smaller changes in Southampton. These findings may reflect the fact that the Cardiff scheme catered more effectively to the needs of walkers and cyclists in providing a safe, convenient and pleasant route, or that the improvements made in Cardiff provided a larger contrast with a lower quality environment prior to the intervention (as indicated by the lower baseline levels of route perceptions among Cardiff residents).

Nonetheless, in Southampton concerns about antisocial behaviour were raised in interviews and the survey data suggested that the infrastructure did not improve residents’ perceptions of safety from crime. Personal safety, which is linked with perceptions of crime, is recognised as influencing walking and cycling, particularly among women [33]. Neither the Kenilworth nor the Southampton schemes were well lit, and both lay on relatively secluded routes separated from commercial and residential areas. Perceptions of personal safety are thought to be influenced by the presence or absence of lighting and by perceptions of ‘surveillance’, a term used to describe the likelihood that a pedestrian or cyclist is observed by others in the area [33]. For example, findings from a cross-sectional study revealed that women preferred to walk along busy roads where they felt less isolated or not to walk at all if the route was deserted [43], while evaluation of a natural experiment revealed that the addition of street lighting led to reductions in crime and fear of crime and an increase in pedestrian street use after dark [44]. It is therefore possible that the lack of lighting and surveillance in Kenilworth and Southampton may have reduced the use of those schemes.

#### Use of new infrastructure may reflect the extent to which it addresses a perceived need

Both qualitative and quantitative data suggested a greater perceived need for the Cardiff scheme than for those in Southampton and Kenilworth. Car journeys in Cardiff were viewed as congested and lengthy, parking at key destinations as difficult and public transport as unreliable, whereas informants at the other two sites did not comment on the challenges faced by users of motor vehicles. Consistent with some travel behaviour theories [45], research suggests that people select their mode of travel according to considerations such as convenience and cost, and therefore that pragmatic factors including access to parking, the reliability of public transport and the relative speed of car travel are important in determining mode choice [46-48]. To some extent our data provide further support to these findings. They suggest that in the context of an environment that is relatively supportive of car travel (as appears to have been the case in Southampton and Kenilworth), the building of safe and pleasant routes for walking and cycling may not be sufficient to drive behaviour change. Conversely, in the context of an environment where motor vehicle use is less attractive (as appears to have been the case in Cardiff), residents may be more likely to use new walking and cycling routes.

Another difference is that in Cardiff, walking and cycling were described as not only unpleasant, but nearly impossible, and to overcome this an entirely new link (a bridge) was added to an existing network of routes. By comparison, the existing environments in Kenilworth and Southampton were more pedestrian-orientated and the infrastructural changes consisted of upgrades of existing networks. Consistent with these views, baseline perceptions of the presence of pavements and the safety of walking were considerably worse in Cardiff than in either Southampton or Kenilworth. A recent mixed-method study of adults who walked or cycled to work despite reporting an unsupportive environment found that they required flexibility in their route choice and typically overcame their unsupportive environments by seeking the least dangerous route [46]. It was acknowledged, however, that to do so required that alternative ‘less dangerous’ routes existed. Although the Connect2 infrastructure in Southampton and Kenilworth may have provided a safer, more convenient route, it may not have provided sufficient contrast to existing routes that pedestrians and cyclists were already willing to use. In the context of Cardiff, however, where the environment was perceived to be more hostile to walking and cycling, the building of new infrastructure may have been sufficient to promote a higher level of use. Having said that, informants acknowledged that an alternative route (the Barrage) existed for pedestrians and cyclists. While this provided a less direct route to the city centre for some, and was perceived by some as unpleasant because of its greater exposure to the elements, pedestrians and cyclists may have continued to take the Barrage route in the absence of adequate feeder routes to the Cardiff Connect2 scheme. Findings from stated preference surveys, although inconsistent, tend to suggest that cyclists are prepared to travel greater distances to use off-road paths and that such facilities are the most important factor in determining route choice [49,50]. This may explain why, despite the greater perceived need in Cardiff, the scheme there was used only marginally more for commuting and other utility journeys than those in Kenilworth and Southampton.

## Conclusion

Drawing on the insights of the realistic approach to evaluation [21] and the heterogeneity of three contrasting case study sites, in this paper we have built on previous work examining the impact of new infrastructure on walking and cycling [25,26] by purposively combining a variety of data sources to explore the mechanisms underlying use of the infrastructure and the contexts which may have enabled or disabled these mechanisms.

We have shown how a variety of data sources can be analysed in combination to enhance our understanding of the mechanisms and conditions leading to the use of new walking and cycling routes. This approach can be particularly insightful in the light of unexpected findings [4] such as the predominance of use for recreation rather than transport. We have argued that this may reflect the need for infrastructural improvements to be coherent and network-wide. Perceptions of the route environment nevertheless improved over time, particularly in Cardiff. This suggests that the schemes were somewhat successful in triggering a key postulated mechanism of action [24], namely improving perceptions of the supportiveness of the environment for walking and cycling — a factor which previous research has shown to be associated with the uptake or maintenance of active travel [51].

Substantial differences in awareness and use between sites were identified, highlighting the potential importance of two additional mechanisms related to the visibility of a scheme. A highly visible scheme may influence not only awareness that the new infrastructure exists, but also social norms related to individual perceptions of how and by whom that infrastructure is being used. It may also influence perceptions of surveillance and safety of the route, which may be important in encouraging or discouraging certain types of use.

Finally, our findings suggest that the differences between sites may also reflect broader contextual differences, in that new walking and cycling infrastructure may be most effective in the context of an environment that is perceived to be hostile to walking and cycling but also unsupportive of car travel. In combination, these analyses have identified several putative mechanisms underlying the effects of environmental interventions on walking and cycling which can be more formally tested in future research.

## Additional file

Additional file 1: **Supplementary material.** Tables S1-S3 and Figures S1-S3.
