# Supplementary material for: Mechanisms underpinning use of new walking and cycling infrastructure in different contexts: mixed-method analysis
Source: Int J Behav Nutr Phys Act. 2015 Feb 21;12:24. doi: 10.1186/s12966-015-0185-5 (PMC4340230; doi:10.1186/s12966-015-0185-5)
Supplement: Supplementary file 1 — Supplementary material. Tables S1-S3 and Figures S1-S3. [file 12966_2015_185_MOESM1_ESM.pdf]

Sahlqvist et al.: Additional File 1

Table S1: Demographic, socio-economic and health characteristics of participants at baseline

Domain	Variable	Level	One-year sample N (%) (N=1849)	Two-year sample N (%) (N=1510)
Geographic	Site	Southampton	523 (28%)	425 (28%)
		Cardiff	596 (32%)	487 (32%)
		Kenilworth	730 (39%)	598 (40%)
	Distance from home to core Connect2 (km)	<1	116 (6%)	92 (6%)
		1-1.99	415 (22%)	329 (22%)
		2-2.99	531 (29%)	447 (30%)
		3-3.99	389 (21%)	324 (22%)
		≥4	398 (22%)	318 (21%)
Demographic	Sex	Female	1006 (54%)	857 (57%)
		Male	843 (46%)	653 (43%)
	Age (years) in 2010	18-34	241 (13%)	144 (10%)
		35-49	379 (21%)	300 (20%)
		50-64	607 (33%)	532 (35%)
		>65	616 (33%)	530 (35%)
	Ethnicity	White	1771 (97%)	1460 (97%)
		Non-White	64 (3%)	45 (3%)
	Any child under 16	No	1547 (84%)	1276 (85%)
		Yes	301 (16%)	234 (16%)
Socio-economic position & car/ bike access	Highest educational Level	Tertiary or equivalent	715 (39%)	590 (39%)
		Secondary school†	619 (34%)	490 (33%)
		None or other	495 (27%)	425 (28%)
	Annual household Income	>£40,000	582 (34%)	451 (32%)
		£20,001-40,000	543 (32%)	469 (33%)
		≤£20,000	565 (33%)	488 (35%)
	Employment Status	Working	938 (51%)	740 (49%)
		Student	48 (3%)	25 (2%)
		Retired	704 (38%)	609 (40%)
		Other	152 (8%)	134 (9%)
	Any car in household	No	247 (13%)	215 (14%)
		Yes	1599 (87%)	1290 (86%)
	Any adult bicycle in household	No	768 (45%)	620 (45%)
		Yes	948 (55%)	768 (55%)

Table S2: Additional informants' quotes illustrating categories

Cardiff	Kenilworth	Southampton
**Expected use and impact**		
**Utility perspective**<br>It will be used by people who commute into the Bay and Cardiff for work and those who want access to the leisure facilities in the Bay. (C1)<br>It will provide four main things: (1) Commuting; even people who get off at Cogan Station having travelled from other areas can then walk across into the Bay; (2) Access to the Sports Village, ice rink, canoe slalom centre; (3) residents going to the bay for shopping…. (C2)<br>Both pedestrians and cyclists will use the bridge and I think we will see an increase in both these groups in terms of the number of people walking or cycling who previously had used other means of transport. (C3)<br>Other users include those who currently use their car on the Cogan Spur and others who can drive or don't have access to a car'. (C3)	**Utility perspective**<br>From the University of Warwick perspective it provides a sustainable transport link from an area where students, staff, researchers etc, live in to the University of Warwick campus. (K3)	**Utility perspective**<br>…..businesses, people going to work, to college, to the university…. Because it connects into the National Cycle Network. (S4)<br>The aim of the project is to encourage people to travel more sustainably, more healthily, cheaper, to reduce car use… (S4)
**Recreation perspective**<br>It will provide four main things..…(4) leisure side e.g., links with the National Cycle Network. (C2)	**Recreation perspective**<br>I think families looking to do off-road cycling, walking, I think the older, I mean the majority of residents in the area are probably 40, 50 plus so they're a little bit older generation. (K3)<br>The Kenilworth greenway…. that's going to be more of a leisure route because you can get to a place called Berkswell, there won't be much traffic coming down from Berkswell. (K5)	**Recreation perspective**<br>I think it will be an attraction for not just local people, but for people coming to do a bit of cycling form further afield. (S3)

Cardiff	Kenilworth	Southampton
**Perceived need for the scheme – utility**		
**Challenges faced by pedestrians and cyclists**<br>Currently the Cogan Spur is the main route to these facilities, and it's not designed for pedestrians and it's difficult for cyclists. (C4)	**Challenges faced by pedestrians and cyclists**<br>I think there will be a migration of existing cycle users from the A429 route to the Connect2 route certainly for getting to the main [university] campus. I'm a pretty experienced cyclist but it's not for the faint hearted along Coventry Road in to Kenilworth, and this gives the option in terms of you know, with two small kids it's the option to take them on bikes, a safe route in to town, but also to the university. (K3)	**Challenges faced by pedestrians and cyclists**<br>…before you had to go through a bit of a winding route… now it's actually going to link up two parts of the city really well to get a bit more of a cross-connection between different areas of the city. (S4)<br>I mean it's quite a frightening place to be on your own at the moment and I think it will open it up and make it much more, um, much nicer to be there... (S4)<br>….there's a gap and that at that point it goes on the road through an industrial estate, it's very dangerous and they're quite well used despite the danger. So I suspect if that piece of cycleway were improved it would be used much more. (S3)
**Existing options for walking and cycling**<br>Although the Barrage opened in June 2008, I think a number of people who currently use it will use the Bridge in preference. The Barrage goes into the east side whereas the Bridge will take people straight into the Bay area. (C3)<br>It [the bridge] will also be a shorter route than the Barrage for accessing Cardiff Bay'. (C2)	No further quotes identified	No further quotes identified
**Challenges faced by other road users**<br>Although it's possible to take a bus, it can take up to 1.5 hours. (C2)<br>Parking is extremely difficult in Cardiff town centre and the Bay. (C2)<br>Lots of commuter routes were appalling for people who lived in Penarth or the Vale of Glamorgan, but worked in Cardiff or Cardiff Bay. (C2)	**Challenges faced by other road users**<br>There's a basic daily permit which is £150 for half a day and then £300 for the day, students are unable, unless they've got special circumstances to buy annual permits now so that have to either by the daily permit. (K3)	**Challenges faced by other road users**<br>Not identified

Cardiff	Kenilworth	Southampton
**Perceived need for the scheme – recreation**		
No further quotes identified	…certainly there are a number of you know, dog walkers, and people looking at alternatives and 'a dry route' rather than a kind of probably inaccessible, muddy single track. (K3)<br>….linking the town with the Countryside. (K4)	No further quotes identified
**Scale of environmental change**		
The road currently from the area to Tesco [almost adjacent to the People's Bridge] is very steep, it's also very narrow. (C1)<br>Busy roads and poor routes on the Vale [of Glamorgan] side leading to the Bridge will mean that it may not be used to its full potential. (C3)	…what it doesn't do is link the university because right through the middle of the university is a road called Gibbet Hill Road and it's just two carriageway but it is quite heavily used. (K5)	No further quotes identified
**Design features of the scheme**		
The paths will be smooth and even, so older people and who are more likely to fall shouldn't have a problem. (C3)<br>We have consulted with the Officer for Disabilities to ensure that the Bridge is wide enough…. It's going to be 4 metres wide. (C4)	…in my mind, rightly or wrongly I see that [the north-south link between Kenilworth and the university] as the core, as the urban link, and I think that's the strength of Connect2 we're trying to encourage… Well, in this case it's a longer than two mile, but those sub two mile journeys that are the major contributor to congestion and CO2 emissions because your car's not warmed up or whatever. (K5)	No further quotes identified

Table S3: Awareness and use of Connect2 infrastructure in 2011, by site, mode and purpose

	**% full sample (N=1826)**	**% Southampton (N=517)**	**% Cardiff (N=589)**	**% Kenilworth (N=720)**
Awareness or use of Connect2	64%	48%	87%	57%
Use of Connect2 (any)	32%	19%	49%	28%
Walking (any)	29%	18%	44%	25%
Transport (any)	11%	8%	19%	7%
Social/leisure	8%	6%	14%	5%
Shopping/personal business	6%	4%	10%	4%
For work	1 (2%†)	1 (1%†)	3 (4%†)	<1 (1%†)
In the course of work	<1 (1%†)	1 (1%†)	1 (1%†)	0 (0%†)
For education	1%	1%	1%	<1%
Recreation	27%	15%	41%	24%
Cycling (any)	13%	7%	19%	12%
Transport (any)	5%	4%	10%	3%
Social/leisure	4%	2%	6%	2%
Shopping/personal business	2%	2%	3%	1%
For work	1 (3%†)	1 (2%†)	4 (6%†)	<1 (1%†)
In the course of work	<1 (1%†)	1 (1%†)	1 (2%†)	0 (0%†)
For education	<1%	<1%	<1%	0%
Recreation	12%	5%	18%	11%
Any walking or cycling	31%	19%	48%	28%
Transport (any)	13%	9%	22%	7%
Social/leisure	9%	7%	16%	5%
Shopping/personal business	6%	4%	11%	4%
For work	2% (4%†)	1% (2%†)	5%	1% (1%)
In the course of work	1% (1%†)	1% (2%†)	1%	0% (0%)
For education	1%	1%	1%	<1%
Recreation	29%	16%	44%	26%

Analyses exclude 1.2% adults with missing 'use' data in 2011
†Percentage of those who reported being in paid employment

Figure S1: ‘Core’ and ‘greater’ Connect2 projects at the Cardiff study site

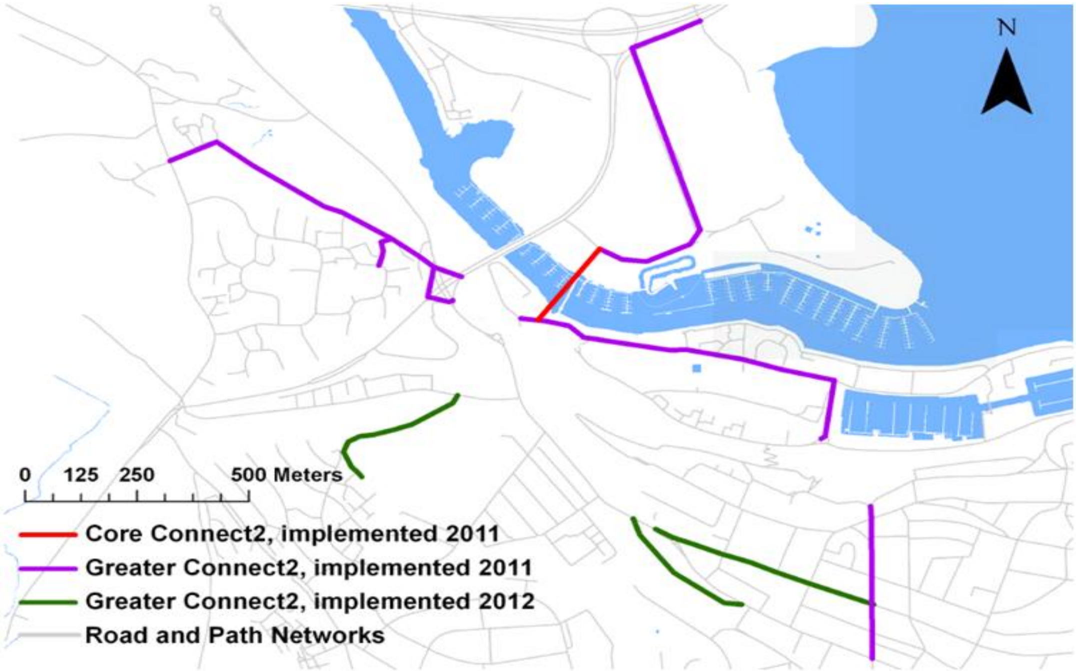

Purple lines show the sections of the greater Connect2 network which were operational at the time of both the 2011 and the 2012 surveys; green lines show the sections of the network only operational at the time of the 2012 survey. Map contains Ordnance Survey data © Crown copyright and database right 2011.

FigureS2: ‘Core’ and ‘greater’ Connect2 projects at the Kenilworth study site

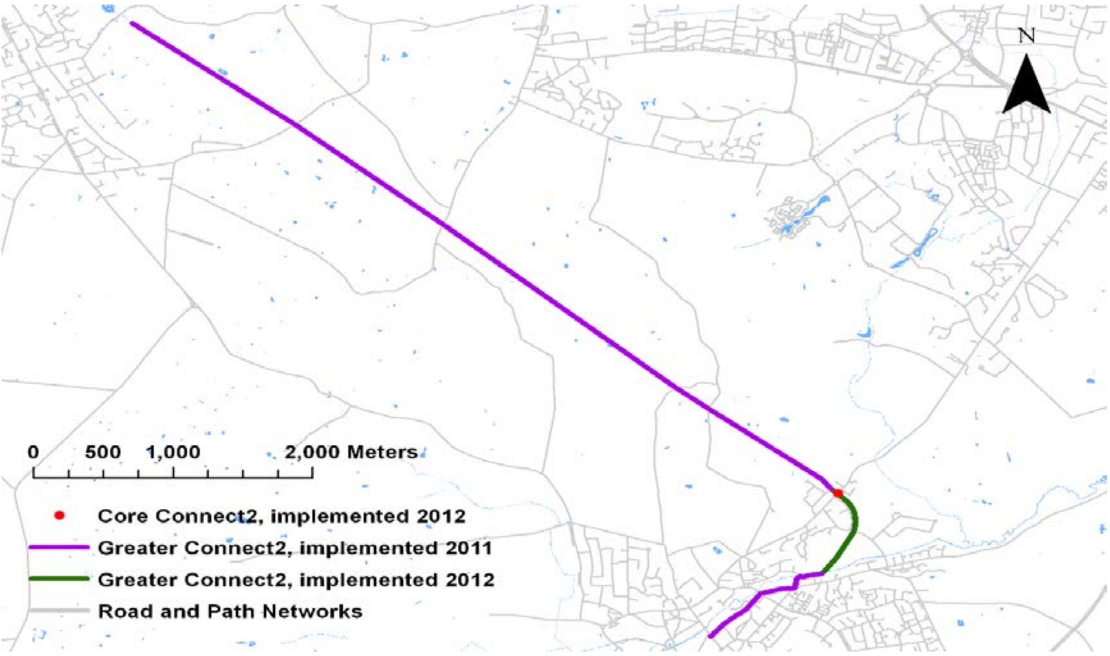

See notes to Figure S1. Map contains Ordnance Survey data © Crown copyright and database right 2011.

Figure S3: ‘Core’ and ‘greater’ Connect2 projects at the Southampton study site

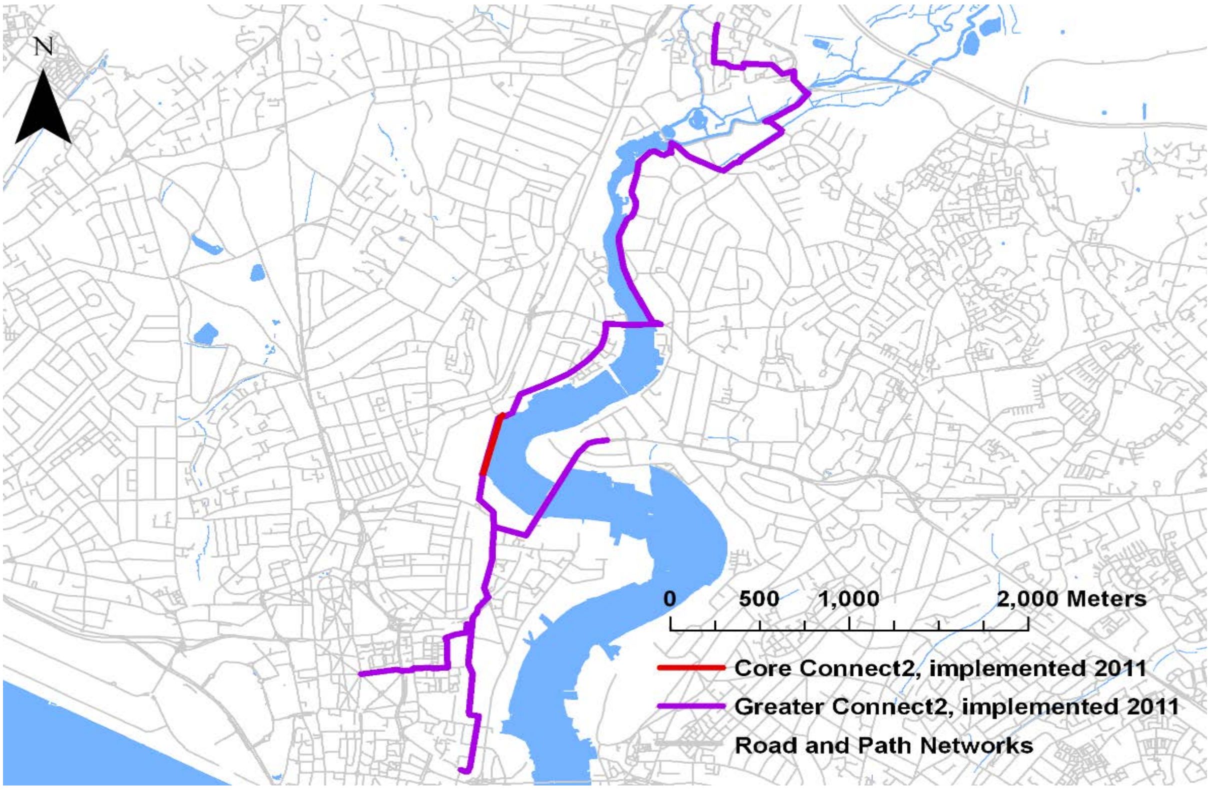

See notes to Figure S1. Map contains Ordnance Survey data © Crown copyright and database right 2011.
